# Energy metabolism in the kidney and its role in chronic kidney disease

**DOI:** 10.1007/s10157-026-02923-9

**Published:** 2026-08-04

**Authors:** Hiroaki Kikuchi, Tomohiro Tsuboya, Jinming Hu, Mari Sugimoto, Miyu Yoshikawa, Maho Usui, Takefumi Suzuki, Mark A. Knepper, Shinichi Uchida, Eisei Sohara

**Affiliations:** 1 Department of Nephrology, Institute of Science Tokyo, Tokyo, Japan; 2 Epithelial Systems Biology Laboratory, National Heart, Lung, and Blood Institute, NIH, Bethesda, MD USA

**Keywords:** Compensatory renal hypertrophy, Chronic kidney disease, AMP-activated protein kinase, unc-51 like autophagy activating kinase 1, Peroxisome proliferator–activated receptor alpha, Proximal tubule, Kidney, Nephron

## Abstract

Chronic kidney disease (CKD) is associated with dysregulated lipid metabolism, particularly in proximal kidney tubules, where fatty acid oxidation serves as the primary energy source. The proximal tubules’ reliance on fatty acid oxidation rather than glycolysis underscores their unique metabolic profile, consistent with the absence of key glycolytic enzymes. This dysregulation contributes to maladaptive hypertrophy in CKD, where surviving nephrons exhibit compensatory hypertrophy to maintain kidney function. Here, we focus on two key regulators of lipid metabolism: peroxisome proliferator–activated receptor alpha (PPARα) and adenosine monophosphate (AMP)-activated protein kinase (AMPK). Recent multi-omics studies have identified PPARα as an important determinant of proximal tubule cell size and a mediator of compensatory hypertrophy. In CKD models, AMPK activity decreases, impairing cellular responses to energy stress, as indicated by altered AMP/ATP ratios. This defective energy sensing may be exacerbated by uremic metabolites that diminish AMPK function. Unc-51-like autophagy activating kinase 1 (ULK1) has been identified as a regulator of AMPK activity through specific phosphorylation sites that enhance AMP sensitivity. Future research should assess whether targeting these pathways restores metabolic homeostasis and mitigates CKD progression by enhancing AMPK activity and lipid metabolism.

##  Background and Introduction

The kidney is one of the most energy-demanding organs, maintaining fluid and electrolyte homeostasis and waste excretion, with kidney blood flow accounting for ~ 20–25% of cardiac output [1]. Fatty acid oxidation (FAO) is the preferred energy source for highly metabolic cells, such as cardiac myocytes, as FAO generates more ATP per molecule than glucose oxidation [2]. Proximal tubular epithelial cells, which reabsorb ~ 70% of the glomerular filtrate, have high baseline energy demands and abundant mitochondria; therefore, the proximal tubule relies primarily on FAO and oxidative phosphorylation rather than glycolysis [3].

FAO involves free fatty acid breakdown in mitochondria and peroxisomes. Although most long-, medium-, and short-chain fatty acids are oxidized in mitochondria, very long-chain fatty acids, fatty dicarboxylic acids, and bile intermediates are oxidized in peroxisomes. Acetyl-CoA carboxylase (ACC), a key enzyme in FAO and fatty acid biosynthesis, has two isoforms: ACC1, abundant in liver and adipose tissue, and ACC2, expressed in highly metabolic organs such as skeletal muscle, heart, and kidney. ACC activity is tightly regulated by 5’-adenosine monophosphate-activated protein kinase (AMPK), sterol regulatory element-binding protein 1a (SREBP1a), SREBP1c, and carbohydrate response element-binding protein. In turn, mitochondrial transcription factors peroxisome proliferator–activated receptor gamma (PPARγ) coactivator 1α and β can stimulate SREBP1a and SREBP1c expression.

Based on data from the Knepper Laboratory at the National Institutes of Health (NIH) (URL: https://esbl.nhlbi.nih.gov/MRECA/Nephron/ ), key glycolytic enzymes—hexokinase (Hk1), phosphofructokinases (*Pfkm*, *Pfkl*, and *Pfkp)*, and pyruvate kinase (*Pkm*)—are minimally expressed in proximal tubules under physiological conditions, supporting the concept that these cells rely predominantly on fatty acid oxidation rather than glucose metabolism for ATP production. Instead, glucose is reabsorbed *via* sodium-glucose cotransporters 1 and 2 and returned to the bloodstream rather than metabolized for energy. This unique metabolic configuration may render proximal tubular cells particularly vulnerable to energetic failure and metabolic stress. Carnitine palmitoyltransferases are also highly expressed in the thick ascending limb and distal convoluted tubule, suggesting that FAO is important in these nephron segments^1^.

## PPARα as a key determinant of kidney hypertrophy

Changes in fatty acid metabolism have received substantial attention, particularly in chronic kidney disease (CKD) and compensatory kidney hypertrophy ([4, 5]). The kidney has a remarkable capacity for hypertrophy. When one kidney is resected—as occurs in kidney transplantation, trauma, or cancer—the contralateral kidney increases in size and function, resulting in functional compensation (compensatory hypertrophy) ([6, 7]). Hypertrophy occurs at the level of individual kidney tubules (nephrons), more than one million of which constitute the kidney parenchyma in humans (210,000 to 2,000,000 nephrons per kidney) [8]. Increased tubule size has been reported in the proximal tubule, distal convoluted tubule, and collecting duct. Nephron hypertrophy also occurs in CKD, where damaged nephrons undergo atrophy while remaining nephrons increase in size and function [9]. This response can blunt the decline in glomerular filtration rate, protecting kidney function but making CKD difficult to detect. Despite many reductionist investigations into the mechanisms of compensatory kidney hypertrophy—including mammalian target of rapamycin ([10, 11]) and growth factor signaling pathways—our knowledge remains incomplete due to the intrinsic complexity of kidney size regulation. However, recent advances in systems biology and “-omics” methodologies have improved the study of such complex processes. The Knepper laboratory applies multiomic approaches—quantitative proteomics, RNA-seq-based transcriptomics, assay of transposase-accessible chromatin sequencing, and phosphoproteomics—to understand kidney pathophysiology, including the syndrome of inappropriate antidiuresis [12] and lithium-induced nephrogenic diabetes insipidus [13].

**Fig. 1:**
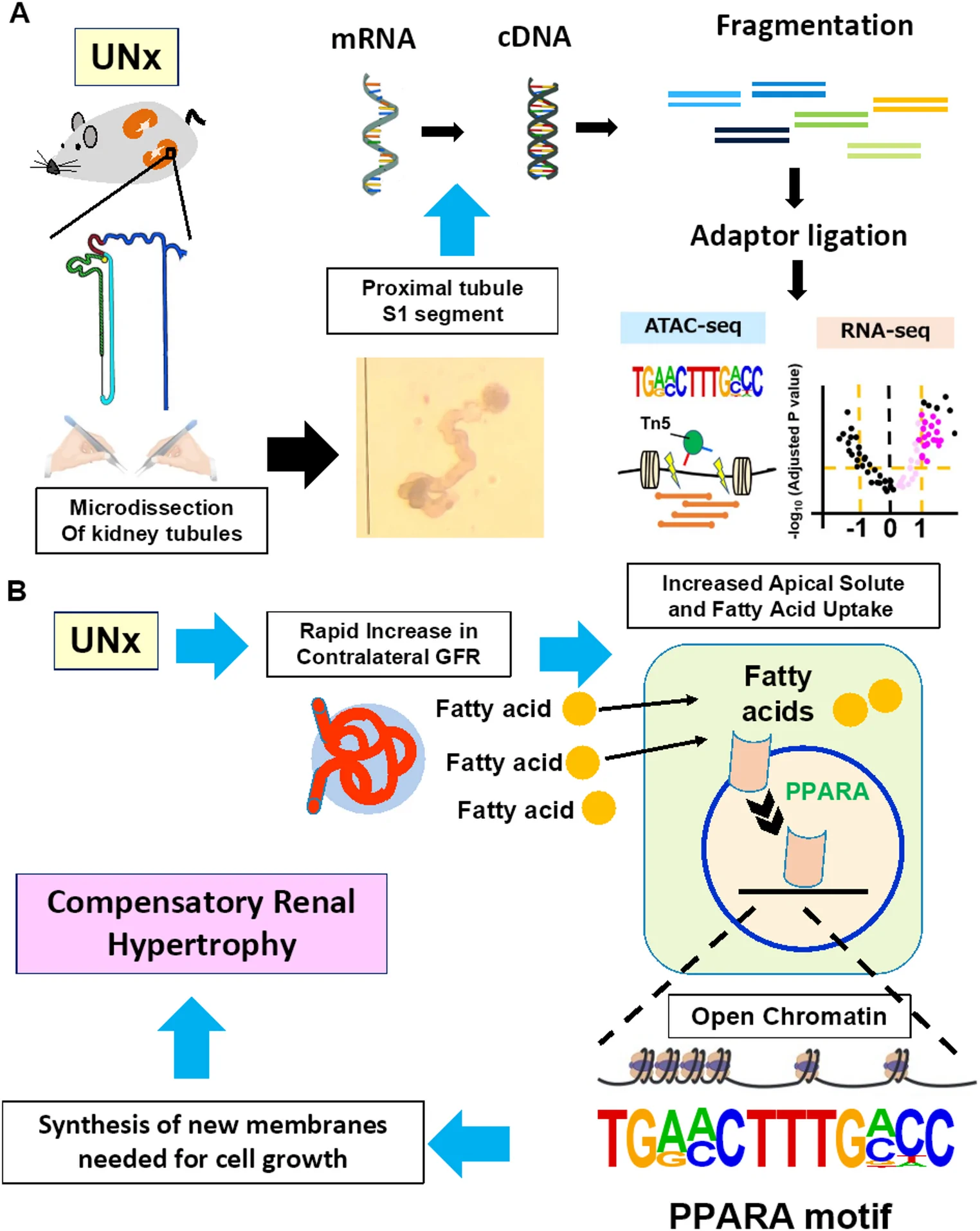
Experimental design and proposed mechanism of fatty acid–PPARα–mediated compensatory renal hypertrophy after UNx. **A** Workflow of transcriptomic and chromatin accessibility analyses in proximal tubular cells after unilateral nephrectomy (UNx). Kidneys from UNx mice were subjected to microdissection to isolate the proximal tubules (S1 segment). mRNA was extracted and converted to cDNA, followed by library preparation through fragmentation and adaptor ligation. RNA sequencing (RNA-seq) and assay for transposase-accessible chromatin using sequencing (ATAC-seq) were performed to assess gene expression and chromatin accessibility, respectively.**B** Proposed model of compensatory renal hypertrophy. UNx induces a rapid increase in contralateral glomerular filtration rate, leading to enhanced apical solute and fatty acid uptake in proximal tubular cells. Increased intracellular fatty acids activate peroxisome proliferator–activated receptor alpha (PPARα), promoting chromatin opening at PPARα motifs and transcriptional programs that enhance fatty acid metabolism and membrane biosynthesis, ultimately driving compensatory renal hypertrophy

In 2023, we sought to identify the earliest signaling changes in the contralateral kidney after unilateral nephrectomy (UNx) in mice using next-generation sequencing and proteomics techniques combined with lipid analysis (Fig. 1) [5]. Kidney volume was already increased 24 h after UNx, reaching a plateau at 72 h. Quantitative morphometry in microdissected proximal tubules showed significant increases in outer diameter and mean cell volume, but no clear increase in the cell count per unit length. Measurements of DNA accessibility (ATAC-seq), transcriptome (RNA-seq) and proteome (quantitative protein mass spectrometry) independently identify patterns of change that are indicative of activation of the lipid-regulated transcription factor, PPARα. Lipid analysis shows an increased abundance of PPARα ligands in the hypertrophied kidney. Activation of PPARα by fenofibrate administration increases proximal tubule cell size, while genetic deletion of PPARα decreases it (Fig. 1). Therefore, we conclude that PPARα plays a critical role in determination of kidney size, a finding that fits with the multi-omics data pointing to a key role for PPARα in compensatory kidney growth.

Cell enlargement requires the production of new cell membranes. The process by which cellular size is increased after PPARα activation is so far unclear. Cell membranes are composed of glycerophospholipids, sphingolipids and sterols [14], as well as cardiolipin in mitochondria [15]. In unilateral nephrectomy, mRNA encoding several lipid synthetic enzymes were increased, especially those regulated by SREBP1, a transcription factor (TF) that controls expression of a wide array of genes involved in biosynthesis of cholesterol and phospholipids including Elovl1 and Cct5 [16]. While PPARα is mostly known for promoting FAO, growing evidence points to its role in regulating lipogenesis dependent on members of the SREBP family [17]. Of particular note, cardiolipin synthase 1 (CRLS1), which is essential for the biosynthesis of the mitochondrial lipid cardiolipin [18], exhibited significantly increased protein levels after UNx at 24 h. The role of PPARα in the production of cellular membranes will require focused studies in the future.

Excess triglyceride accumulation can induce cellular lipotoxicity and may contribute to kidney disease irrespective of systemic hyperlipidemia ([4, 19]). Although the healthy kidney has a relatively low lipid content, lipid accumulation occurs early in CKD and promotes disease progression [4]. Clinical and experimental studies demonstrate that kidney lipid accumulation occurs independently of hyperlipidemia in diabetic kidney disease, hypertensive nephrosclerosis [20], focal segmental glomerulosclerosis [21], minimal change disease [22] and Alport syndrome [23]. Thus, restoring FAO by targeting relevant signaling pathways may help ameliorate CKD progression.

## AMPK and its regulation

The energy sensor AMPK is an evolutionarily conserved serine/threonine kinase. Its activity is highly sensitive to cellular energy stress, reflected by increased AMP levels and a higher AMP/ATP ratio, making AMPK a key regulator of cellular energy status [24]. To maintain energy homeostasis, AMPK activates pathways that promote ATP production [25] AMPK consists of three subunits—α, β, and γ—with the α-subunit containing the catalytic domain [26]. Binding of AMP and/or adenosine diphosphate (ADP) to the γ-subunit induces a conformational change in the β-subunit [27], facilitating phosphorylation at Thr172 in the α-subunit by AMPKs such as liver kinase B1 (LKB1) and Ca²⁺/calmodulin-dependent protein kinase kinase 2 [28], resulting in AMPK activation. In this way, AMPK enables cells to adapt to changes in cellular energy demand. Beyond energy metabolism, AMPK also regulates cell proliferation, apoptosis, and inflammation ([29–32]).The γ-subunit consists of two Bateman domains, each containing two cystathionine β-synthase (CBS) repeats. These four CBS repeats form four potential ligand-binding sites in AMPK that can bind AMP, ADP, or ATP competitively. ATP competitively inhibits AMP and ADP binding to the γ-subunit, making AMPK a sensor of AMP/ATP or ADP/ATP ratios. Under energy-replete conditions (low AMP/ATP and ADP/ATP ratios), phosphatases can access Thr172 to keep AMPK unphosphorylated. When energy is depleted, elevated AMP and ADP bind to CBS3, of the AMPK γ-subunit, preventing phosphatase access and enhancing α-subunit phosphorylation. AMP (and to a lesser extent ADP) binding to CBS3 also stimulates LKB1-mediated phosphorylation, which requires myristoylation of the β-subunit [33] Additionally, AMP binding to CBS1 allosterically increases intrinsic AMPK activity. Quantitatively, activation by phosphorylation is far greater (> 1000-fold) than allosteric activation (up to fivefold) [34].

## Physiological roles of AMPK in the kidney

AMPK is also potentially an important regulator energy metabolism in the kidney. AMPK has been reported to have roles in ion transport [35], podocyte function [36], ischemia [37], inflammation [38], and diabetes ([39, 40]). Interestingly, inhibition of AMPK is also involved in renal hypertrophy induced by diabetes [40]. Also, several studies reported the mechanisms by which reduced AMPK activity in the diabetic kidney has been linked to renal hypertrophy, including increased triglyceride accumulation because of reduced inhibitory phosphorylation of acetyl-CoA carboxylase ([37, 41–43]) and increased high glucose-induced protein synthesis [40]. Given that renal diabetes-induced hypertrophy is strongly correlated with worse renal outcome ([44, 45]), renal hypertrophy induced by decreased AMPK activation might be associated with renal prognosis.

Between 2010 and 2020, reduced renal AMPK activity has been reported to be associated with impaired renal function and inflammation in the kidneys of diabetic patients [46], obesity-associated nephropathy [47] and experimental CKD models ([48, 49]). Consequently, AMPK has emerged as a novel therapeutic target for CKD. However, until now, the mechanisms underlying AMPK dysregulation in CKD, and whether activation of AMPK is renoprotective or potentially harmful, remain unclear.

## AMPK sensing failure to AMP/ATP ratio in the CKD kidney

To understand the mechanism by which AMPK is suppressed in the CKD kidney, the levels of AMP, ADP, and ATP in the kidney were assessed. We performed metabolomic analysis using capillary electrophoresis–time-of-flight mass spectrometry in the subtotal nephrectomy (5/6 Nx) kidney model, in which no severe renal cellular morphological changes were observed [50, 51].

Although subtle histological changes were observed 8 weeks after 5/6 Nx, principal component analysis revealed remarkable differences in metabolomic profiles between sham-control and 5/6 Nx mice. In addition, the levels of phosphorylated ACC and phosphorylated unc-51-like autophagy activating kinase 1 (Ulk1) were decreased in the kidneys of 5/6 Nx mice, suggesting suppressed AMPK activity.

**Fig. 2:**
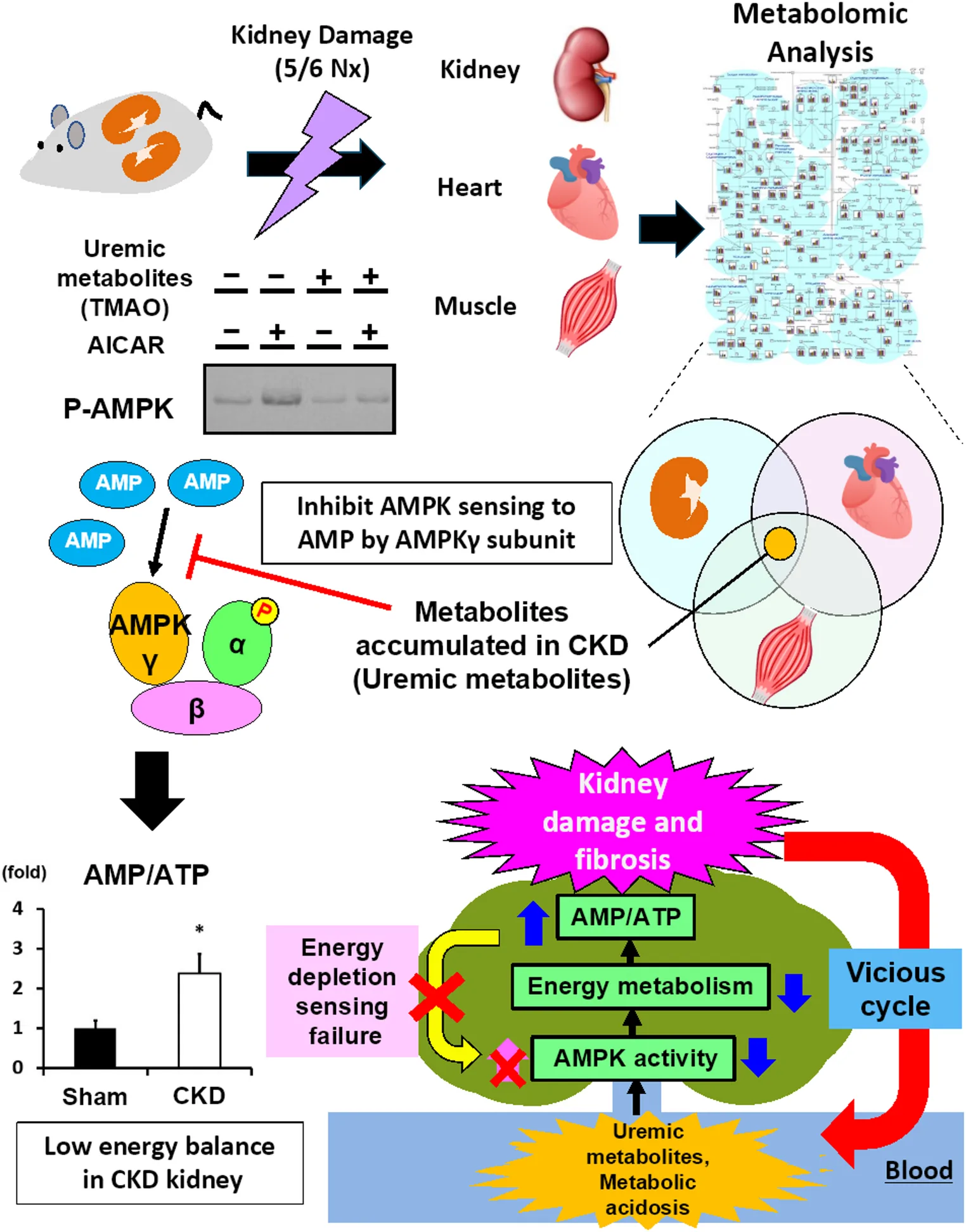
Metabolomic identification of uremic metabolites and impaired AMPK-dependent energy sensing in chronic kidney disease. Kidney injury was induced by 5/6 nephrectomy (5/6 Nx), followed by metabolomic analyses of the kidney, heart, and skeletal muscle to identify systemic metabolic alterations. Metabolites commonly accumulated across multiple organs inhibit AMP sensing by the γ subunit of AMP-activated protein kinase, thereby suppressing AMPK activation. Impaired AMPK signaling disrupts cellular energy homeostasis, resulting in defective energy depletion sensing, reduced energy metabolism, and decreased AMPK activity despite an increased AMP/ATP ratio observed in CKD kidneys. This metabolic mismatch leads to low energy balance, promoting kidney damage and fibrosis. Accumulation of uremic metabolites further exacerbates metabolic acidosis and AMPK dysfunction, forming a vicious cycle that accelerates CKD progression. 5/6Nx, subtotal nephrectomy; TMAO, trimethylamine N-oxide; AICAR, 5-aminoimidazole-4-carboxyamide ribonucleoside

As AMPK is typically inactivated under energy-replete conditions (i.e., low AMP/ATP ratio, section. 3), we next assessed the AMP/ATP ratio in the kidney using capillary electrophoresis–time-of-flight mass spectrometry. However, unexpectedly, the kidneys of 5/6 Nx mice exhibited lower ATP levels and higher AMP levels than those of sham controls, indicating a failure of AMPK to properly sense the AMP/ATP ratio (Fig. 2).

Furthermore, the level of phosphorylated LKB1 at Ser428 (P-LKB1), an upstream kinase that phosphorylates Thr172 of AMPK, was not decreased in the kidneys of 5/6 Nx mice. This physiological phenomenon—impaired AMPK sensing of the AMP/ATP ratio—was first identified in our study. To elucidate the mechanism underlying this sensing failure, we investigated whether it is induced by local factors, such as hypertrophic responses in the remnant kidney, or by systemic factors, such as uremic metabolites.

To determine whether AMPK dysregulation in 5/6 Nx kidneys is caused by systemic or local factors, we compared P-AMPK/total AMPK status between the sham right intact kidney and the left incised remnant (2/6 Nx) kidney of sham-control mice. No significant change was observed between the sham intact kidney and the left 2/6 Nx, suggesting that the change is not caused by local effects of surgery but by systemic factors linked to CKD status.

From metabolomics analysis, several metabolites were found to accumulate systemically in 5/6 Nx mice, including indoxyl 3-sulfate, trimethylamine N-oxide (TMAO), creatinine, allantoin, 6-aminohexanoic acid, and asymmetric dimethylarginine. Among these, indoxyl 3-sulfate and trimethylamine N-oxide attenuated 5-aminoimidazole-4-carboxamide ribonucleotide (AICAR) -induced AMPK activation in HK-2 cells, a human proximal tubular epithelial cell line. These findings suggest that uremic conditions impair AMPK sensing of the intracellular AMP/ATP ratio (Fig. 2).

Based on these observations, we next sought to elucidate the molecular mechanisms underlying defective AMP sensing by the AMPK γ-subunit in CKD, focusing in particular on posttranslational modifications of the AMPK γ-subunit as a potential regulatory mechanism.

## ULK1 regulates AMP sensitivity in AMPK

**Fig. 3:**
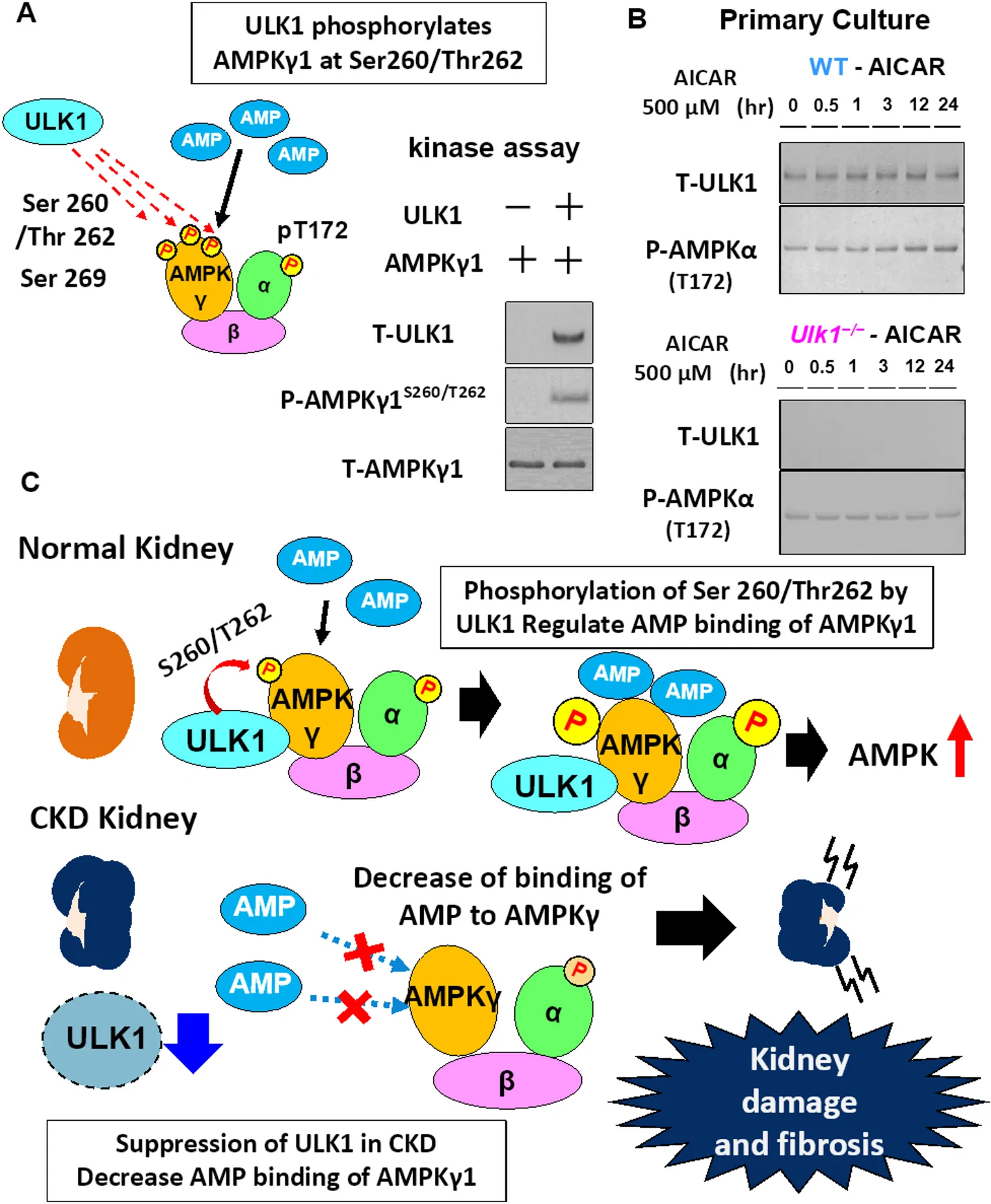
ULK1-dependent phosphorylation of AMPKγ1 regulates AMP sensing and is impaired in chronic kidney disease. **A** Schematic illustration of the molecular interaction between ULK1 and AMP-activated protein kinase (AMP-activated protein kinase, AMPK). ULK1 phosphorylates the γ1 subunit of AMPK at Ser260, Thr262, and Ser269. Immunoblot analysis showed total ULK1 (T-ULK1), phosphorylated AMPKγ1 at Ser260/Thr262 (P-AMPKγ1 ^S260/T262^), and total AMPKγ1 (T-AMPKγ1), confirming ULK1-dependent phosphorylation of AMPKγ1. **B** Immunoblots of phospho-Thr172 AMPK (P-AMPK) and total ULK1 (T-ULK1) in primary cultures of kidney proximal tubule cells obtained from both WT mice (up) and ULK1^-/-^ mice (bottom). Cultured cells were treated with AICAR (500µM) for 0.5 h, 1 h, 3 h, 12 h, or 24 h. **C** Proposed model of ULK1-mediated regulation of AMPK activity in normal and CKD kidneys. In normal kidneys, ULK1 phosphorylates AMPKγ1, facilitating AMP binding to the γ subunit and promoting AMPK activation. In chronic kidney disease (CKD), suppression of ULK1 reduces phosphorylation of AMPKγ1, leading to decreased AMP binding and impaired AMPK activation. Defective AMP sensing compromises cellular energy homeostasis and causes kidney injury and fibrosis

In 2011, Loffer et al. used liquid chromatography–tandem mass spectrometry to identify interacting proteins and substrates of ULK1 [52]. They found that all AMPK subunits are phosphorylated by ULK1, suggesting a bidirectional regulatory relationship between ULK1 and AMPK (Fig. 3A).

Phosphorylation sites in the AMPK γ-subunit (Ser260, Thr262, and Ser269) lie in the linker region between CBS motifs 3 and 4, part of an AMP/ATP-binding Bateman domain. Because this region senses nucleotides (Section. 3), phosphorylation may modulate AMP/ATP binding and AMPK activity, though this was not initially investigated.

In 2024, using cultured primary cells from wild-type and Ulk1^-/-^ mice, the 5-aminoimidazole-4-carboxamide ribonucleotide (AICAR) treatment-induced increase in Thr172 phosphorylation of AMPK was abolished in ULK1-deficient cells, suggesting ULK1’s role in AMP sensing (Fig. 3B). Kinase assays with recombinant ULK1 and trimeric AMPK α1β1γ1 confirmed direct phosphorylation of AMPK γ1 at the Ser260 and Thr262 sites by ULK1. To investigate whether this phosphorylation promotes AMPK activity, mutants with AMPK γ1^Ser260/Thr262^ replaced by alanine were created. In these mutants, Thr172 phosphorylation of the α-subunit by the selective ULK1 agonist BL918 [53] was abolished, demonstrating that ULK1 facilitates AMPK α activation *via* AMPK γ1 Ser260/Thr262 phosphorylation.

Next, we investigate whether AMPK γ1^Ser260/Thr262^ phosphorylation directly regulates AMP binding to the CBS domains. To test this, we generated trimeric AMPK α1^WT^ β1^WT^ γ1^WT^ and AMPK α1^WT^ β1^WT^ γ1^S260A/T262A^ mutant protein complexes (Fig. 3) and co-incubated them with ULK1. AMP binding was assessed using the fluorescent probe Mant-AMP. Recombinant AMPK γ1 phosphorylation at Ser260/Thr262 by ULK1 significantly increased AMPK binding to AMP compared with controls, whereas AMP binding to AMPK α1β1γ1^S260A/T262A^ mutant was significantly lower than that to the AMPK α1β1γ1^WT^, suggesting that phosphorylation at these sites is critical for AMP binding to the AMPK γ-subunit.

Overall, our study revealed that ULK1 promotes cellular AMP sensing by AMPK through phosphorylation of Ser260/Thr262 on the AMPK γ1-subunit [54]. Given that AMP-sensing failure was observed in CKD kidneys, ULK1 activation might represent a novel therapeutic approach for CKD by improving AMPK’s AMP sensitivity (Fig. 3C). Although the ULK1 agonist BL918 activated AMPK in NRK-52E cells, ULK1 activation in kidneys in vivo was not confirmed, likely due to limited distribution of BL918. Future studies are warranted to evaluate more efficient ULK1 activators and their effects on CKD progression.

## Conclusion and future directions

In this review, we summarize the emerging concept of metabolic reprogramming in the kidney, with particular emphasis on the ULK1/AMPK and PPARα signaling axes. Accumulating evidence indicates that restoration of FAO through activation of these pathways confers substantial renoprotective effects, consistent with the fundamental dependence of proximal tubular cells on mitochondrial FAO for energy homeostasis.

However, this therapeutic strategy warrants caution. In clinical settings, excessive catabolism is often accompanied by elevated blood urea nitrogen, suggesting that indiscriminate or sustained FAO activation may impose metabolic stress rather than protection. Thus, the relationship between FAO activation and renal health is not simply linear but likely follows a narrow therapeutic window. From a metabolic perspective, continuous AMPK activation by AICAR may therefore not represent the optimal strategy for slowing CKD progression. In contrast, ULK1 may act as a context-dependent regulator of AMPK activity by modulating AMP sensing through phosphorylation of the AMPK γ1 subunit. Such a mechanism would allow AMPK activation to be enhanced preferentially under conditions of energetic stress, while avoiding excessive activation when cellular energy demand is low. Therefore, therapeutic strategies targeting the ULK1–AMPK axis may provide a more physiologically balanced approach to preventing CKD progression than constitutive pharmacological AMPK activation.

Future efforts should move beyond simple pathway activation toward a nuanced understanding of metabolic quality control. Integrative multiomic approaches—including metabolomics, flux analysis, and single-cell transcriptomics—will be essential to distinguish adaptive AMPK-mediated responses from maladaptive ones in proximal tubular cells.

Importantly, impaired energy sensing—specifically, AMPK’s failure to appropriately respond to changes in the AMP/ATP ratio—may be a key driver of CKD progression. Rather than globally stimulating catabolism, restoring this intrinsic metabolic sensing may offer a safer, more physiologically aligned therapeutic strategy.

Taken together, targeting metabolic resilience, rather than mere activation, could define the next generation of CKD treatments.

## Data Availability

Not applicable.

## Footnotes

1 Non-italicized gene symbols are used to designate proteins or transcripts. When referring specifically to the gene rather than its gene products, gene symbols are italicized. Official gene symbols are archived by the *Nomenclature Committee of the Human Genome Organization* (HUGO) and are accessible at https://www.genenames.org/.
